# The biotechnological potential of the *Chloroflexota* phylum

**DOI:** 10.1128/aem.01756-23

**Published:** 2024-05-06

**Authors:** André Freches, Joana Costa Fradinho

**Affiliations:** 1 Associate Laboratory i4HB - Institute for Health and Bioeconomy, NOVA School of Science and Technology, NOVA University of Lisbon, Caparica, Portugal; 2 Department of Chemistry, UCIBIO - Applied Molecular Biosciences Unit, NOVA School of Science and Technology, NOVA University Lisbon, Caparica, Portugal; Kyoto University, Kyoto, Japan

**Keywords:** filamentous anoxygenic phototroph, photoautotrophic bacteria, extremophiles, biotechnological applications, decontamination technologies, value-added substances production

## Abstract

In the next decades, the increasing material and energetic demand to support population growth and higher standards of living will amplify the current pressures on ecosystems and will call for greater investments in infrastructures and modern technologies. A valid approach to overcome such future challenges is the employment of sustainable bio-based technologies that explore the metabolic richness of microorganisms. Collectively, the metabolic capabilities of *Chloroflexota*, spanning aerobic and anaerobic conditions, thermophilic adaptability, anoxygenic photosynthesis, and utilization of toxic compounds as electron acceptors, underscore the phylum’s resilience and ecological significance. These diverse metabolic strategies, driven by the interplay between temperature, oxygen availability, and energy metabolism, exemplify the complex adaptations that enabled *Chloroflexota* to colonize a wide range of ecological niches. In demonstrating the metabolic richness of the *Chloroflexota* phylum, specific members exemplify the diverse capabilities of these microorganisms: *Chloroflexus aurantiacus* showcases adaptability through its thermophilic and phototrophic growth, whereas members of the *Anaerolineae* class are known for their role in the degradation of complex organic compounds, contributing significantly to the carbon cycle in anaerobic environments, highlighting the phylum’s potential for biotechnological exploitation in varying environmental conditions. In this context, the metabolic diversity of *Chloroflexota* must be considered a promising asset for a large range of applications. Currently, this bacterial phylum is organized into eight classes possessing different metabolic strategies to survive and thrive in a wide variety of extreme environments. This review correlates the ecological role of *Chloroflexota* in such environments with the potential application of their metabolisms in biotechnological approaches.

## INTRODUCTION

The utilization of microorganisms in biotechnological processes has been widely reported, and the search for new metabolisms can lead to the development of several innovative technologies focused on environmental decontamination, wastewater treatment, and production of energy and value-added substances (1–4). In all these applications, the adaptability and versatility of microorganisms make them valuable tools for addressing environmental and industrial challenges. In every microbial phylum, we can find interesting metabolisms, and in the specific case of the *Chloroflexota* phylum, it encompasses a wide spectrum of metabolic diversity, with some organisms exhibiting remarkable traits such as a bicycle-like mechanism for inorganic carbon fixation, others harnessing the power of halogens, and others performing denitrification, although often being involved in the cycling of several elements (5). This diversity, which arises from the natural adaptation of *Chloroflexota* to harsh environmental conditions, contains a unique array of metabolic processes and distinctive features that can be explored for biotechnological purposes.

**Fig 1:**
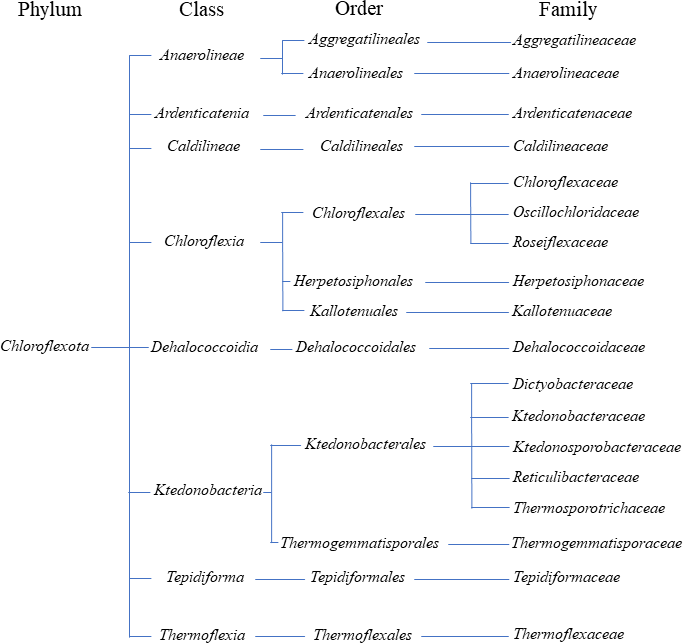
The *Chloroflexota* phylum is divided into eight classes of bacteria, each with its specific orders and families (https://lpsn.dsmz.de/phylum/chloroflexota, accessed 10 February 2024).

The *Chloroflexota* bacteria phylum, formerly known as green non-sulfur bacteria (GNSB), comprises extensively diverse microorganisms that can be found in several environments, both terrestrial and aquatic. This phylum nomenclature derives from the species *Chloroflexus aurantiacus*, first isolated and described by Pierson and Castenholtz (6) as a filamentous anoxygenic phototroph (FAP), a term currently used specifically for phototrophic members of the *Chloroflexota*. The constant discoveries of new microbial organisms and advances in phylogenetic analysis are leading to a continual redefinition and reorganization of this phylum. Currently, the *Chloroflexota* phylum is divided into eight classes: *Anaerolineae*, *Ardenticatenia*, *Caldilineae*, *Chloroflexia*, *Dehalococcoidia*, *Ktedonobacteria*, *Tepidiformia*, and *Thermoflexia* (Fig. 1). Interestingly, the entire phylum only has 18 families, with many of the classes owing their name to one single genus characteristic of the class.

The uniqueness of *Chloroflexota* members is reviewed in this manuscript, analyzing its metabolic features and placing a great focus on the much-needed overview of the biotechnological potential of this bacterial phylum.

## *CHLOROFLEXOTA* CLASSES AND CHARACTERISTICS

**Fig 2:**
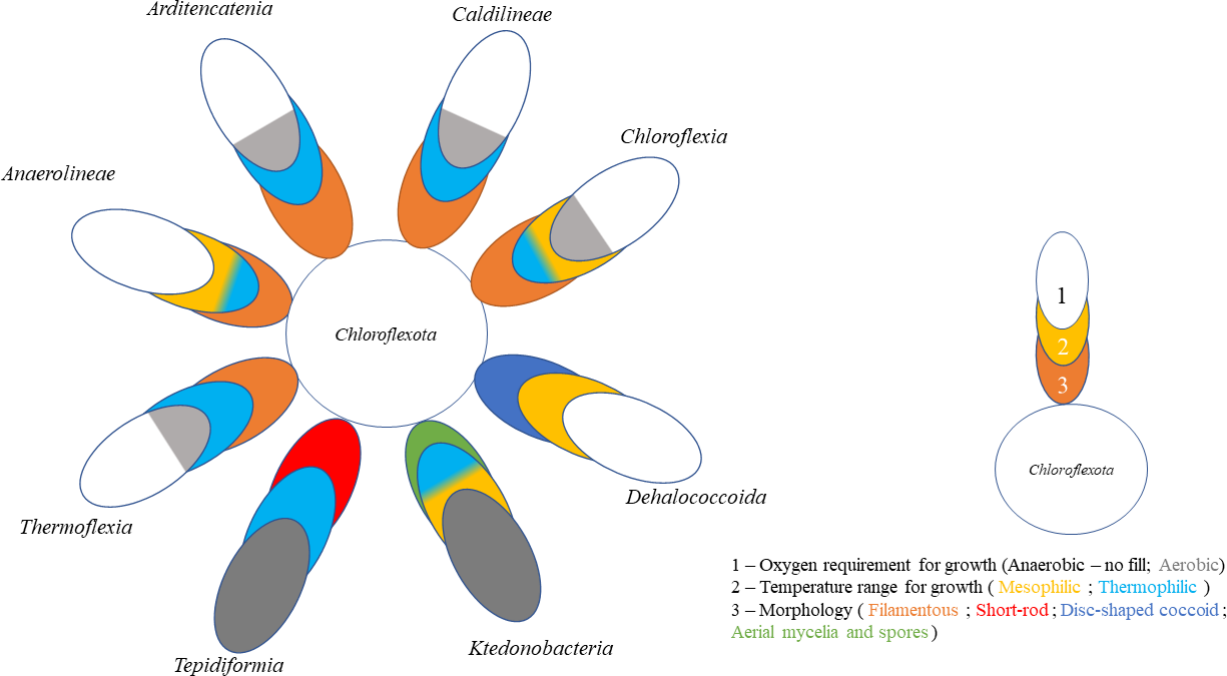
The *Chloroflexota* phylum organized in terms of predominant morphology, oxygen requirements, and temperature range for growth (mesophiles: 20°C–45°C; thermophiles: 45°C–80°C).

The *Chloroflexota* phylum is characterized according to the metabolism, phylogeny, cell shape, motility, ability to form multicellular aggregates, and spore formation capacity. There are several similar traits (morphology, environment, and growth conditions) between the constituents of this bacterial phylum, as shown in Fig. 2.

The class *Anaerolineae* is divided into two orders, *Aggregatilineales* and *Anaerolineales*, each containing a single family. Its representatives are strictly anaerobic chemoorganotrophic organisms with a filamentous morphology. Organisms from this class have been isolated from diverse environments including anaerobic digesters, hot springs, and sub-seafloor sediments (7, 8).

The class *Ardenticatenia* was proposed based on a single species, *Ardenticatena maritima,* which was isolated from a costal hydrothermal field, and is a facultative aerobe that can reduce ferric ion and nitrate under anaerobic conditions. This chemoorganotrophic species forms thin multicellular filaments and can grow at temperatures of up to 75°C (9).

The class *Caldilineae* contains a single described order, *Caldilineales*, and family, *Caldilineaceae*, which consists of two genera, *Caldilinea* and *Litorilinea*. Species belonging to these genera are aerobic, facultative aerobic, or anaerobic chemoorganotrophic filamentous organisms found in hot springs and hot aquifers (7, 10, 11).

The class *Chloroflexia* contains the first known members of this phylum that possess phototrophic and/or chemoheterotrophic growth under mesophilic or moderately thermophilic conditions, presenting a filamentous growth morphology. Moreover, the phototrophic members of this class, usually referred to as FAP bacteria, belong to the *Chloroflexales* order, which can be divided into three families: *Chloroflexaceae* (photoheterotrophic but also with photoautotrophic abilities), *Roseiflexaceae,* and *Oscillochloridaceae* (both predominantly photoheterotrophic). The first two families are composed of thermophilic FAP bacteria isolated from terrestrial hot springs, whereas the latter contains photoheterotrophic mesophilic freshwater species (12, 13). Although phototrophy in *Chloroflexota* isolates is limited to *Chloroflexia* class, metagenomic studies indicate the existence of potentially phototrophic members also in other classes, which could be attributed to horizontal gene transfer of sequences for reaction center and bacteriochlorophyll synthesis proteins (14). The *Chloroflexia* class has two additional orders, *Herpetosiphonales* and *Kallotenuales*, containing non-photosynthetic species that rely on heterotrophic metabolism for growth (12, 15).

The class *Dehalococcoidia* is characterized by the disc-shaped coccoid form of its members, instead of the filamentous morphology attributed to other classes within the *Chloroflexota* phylum. All isolates from this class can grow in chemoorganotrophic conditions and perform dehalogenation of chlorinated and brominated alkanes under strict anaerobic conditions, which grants them great importance in the bioremediation field (16).

The class *Ktedonobacteria* contains heterotrophic bacteria capable of growing under microaerophilic conditions. They have been isolated from soil samples with the peculiarity of forming aerial mycelia and spores. This class is divided into two orders, *Ktedonobacterales* and *Thermogemmatisporales*. The order *Ktedonobacterales* contains all the mesophilic and some thermophilic representatives of this class, whereas the *Thermogemmatisporales* order encompasses only thermophilic species (17–22).

The class *Tepidiformia* is composed of one order, *Tepidiformales*, and one family, *Tepidiformaceae*, containing moderately thermophilic bacteria with a regular short rod morphology, being able to grow hetero- or auto-trophically in aerobic conditions (23).

The class *Thermoflexia*, proposed based on its type species *Thermoflexus hugenholtzii*, englobes filamentous thermophilic chemoheterotrophic microaerobes (optimally growing at 1% (vol/vol) O_2_ with an upper limit of 8% O_2_) being also facultatively anaerobic (24).

## *CHLOROFLEXOTA* METABOLISMS AND ENVIRONMENTS

**Fig 3:**
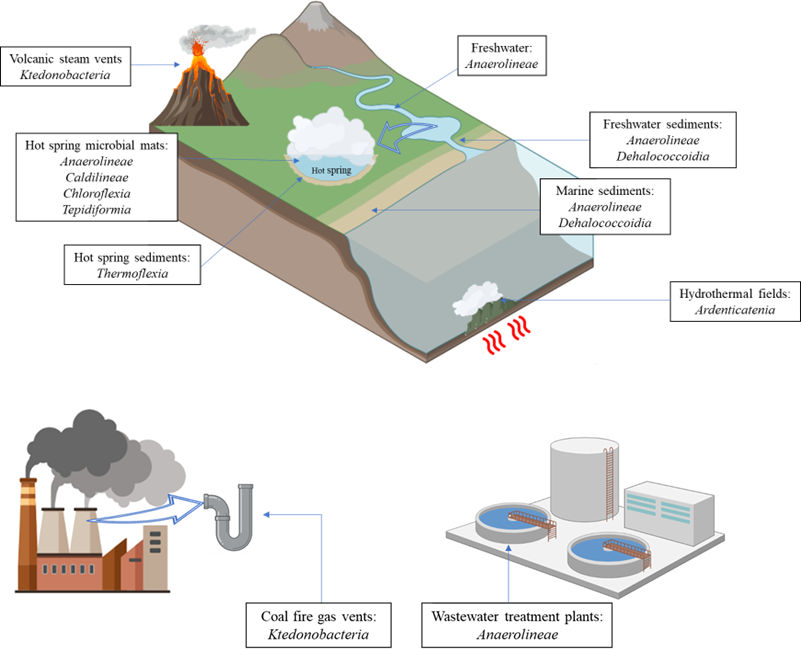
Natural and industrialized environments where *Chloroflexota* can be found. The pictures were designed with templates available at https://www.biorender.com/.

There are several metabolisms present within the *Chloroﬂexota* phylum, such as anoxygenic phototrophy, obligate anaerobic heterotrophy, organohalide respiration, and facultative or aerobic heterotrophy. For this reason, bacteria from the *Chloroflexota* phylum can be found in several environments (natural or industrial) with different properties and singularities (25–29). Their metabolic versatility allowed the adaptation of *Chloroflexota* to microbial mats, soils, aquatic environments, and other extreme environments as shown in Fig. 3.

### Anoxygenic phototrophy

The ﬁrst metabolism to be described in this phylum was anoxygenic phototrophic growth (6), in which the ﬁlamentous phototrophs use light energy to generate chemical energy in the form of ATP. The isolated members from the *Chloroflexota* phylum exhibiting photoautotrophy are exclusively contained in the *Chloroflexia* class; however, most organisms belonging to this class demonstrated the ability to grow photoheterotrophically assimilating organic carbon compounds (30–33), or chemotrophically under aerobic dark conditions. *Chloroflexia* is commonly found in phototrophic microbial mat communities, specifically in neutral and alkaline spring waters with temperatures between 40°C and 70°C (6, 34–36).

To perform their main metabolism, the phototrophic bacteria rely on light-harvesting organelles known as chlorosomes, oval structures attached to the inner surface of the cytoplasmic membrane, consisting of paracrystalline aggregates of bacteriochlorophylls that are surrounded by a galactolipid non-unit membrane (37). Regarding the antenna pigments, there are some slight differences within the order *Chloroflexales*: for example, members of the *Chloroflexus* and *Oscillochloris* genera possess bacteriochlorophyll *c*, whereas *Chloronema* is rich in bacteriochlorophylls *c* and *d* (31, 32). The presence of bacteriochlorophylls *c* or *d* gives cells their green color, and carotenoids (β- and γ-carotene, and derivatives) are also present as part of the light-harvesting cellular apparatus. *Roseiflexus castenholzii* and *Heliothrix oregonensis* (from the family *Roseiflexaceae*) contain solely bacteriochlorophyll *a*, which makes them chlorosome-free. These cells usually present a red/orange color because of their oxo-γ-carotene and glucoside-rich nature. Moreover, the carotenoid β-carotene (which is present in *Chloroflexus*, *Oscillochloris*, and *Chloronema*) is missing in the red-colored genera *Roseiflexus* and *Heliothrix* (38–40).

In terms of inorganic carbon fixation, *C. aurantiacus* can use the 3-hydropropionate bi-cycle, in which bicarbonate fixation is proceeded by the carboxylation of acetyl-CoA and propionyl-CoA yielding pyruvate as the net product; glyoxylate, an intermediate of the bi-cycle, can also be assimilated into cell material (41–44). Energy-wise, inorganic carbon fixation via the 3-hydroxypropionate bi-cycle requires 7 ATP, 5 NAD(P)H, and 3 HCO_3_^−^ to produce one pyruvate. Comparatively, alternative carbon fixation pathways found in other phototrophs can produce pyruvate through the reverse tricarboxylic cycle, which requires, equivalently, 3 CO_2_, 2 ATP, and 5 NAD(P)H, whereas the Calvin-Benson-Bassham cycle inputs 3 CO_2_, 7 ATP, and 5 NAD(P)H to produce one pyruvate molecule (45, 46). However, the 3-hydroxypropionate bi-cycle allows the consumption of bicarbonate, which can provide a competitive advantage to *Chloroflexus* in alkaline environments or under low carbon dioxide availability (47). To support inorganic carbon fixation, different compounds can be used as electron sources, such as sulfide, thiosulfate, small organic molecules, or molecular hydrogen (45, 48–50).

Given the ability to use a wide range of electron donors, members of the *Chloroflexia* class (specifically members of the *Chloroflexales* order) have also been observed in marine and hypersaline microbial mats (51, 52), which points to the adaptability of such organisms to different environments. Indeed, the presence of *Chloroflexales* members has also been reported in extreme soil environments such as sediments from the Arctic (53). Furthermore, *Chloroflexia* has been isolated from volcanic vents (54), and *Chloroflexus islandicus* has been isolated from a geyser in Iceland (30). Ecologically, *Chloroflexia* serves as both primary and secondary producers (55–57), performing mixotrophic growth using CO_2_ and simple organic molecules as carbon sources.

### Anaerobic heterotrophy

Strict anaerobic chemoheterotrophic growth has been reported in the classes *Anaerolineae* and *Dehalococcoidia*. The strict anaerobic metabolisms of *Anaerolineae* are characterized by a fermentative metabolism, with some isolates performing sugar fermentation to produce acetate, lactate, succinate, propionate, and hydrogen and this class has shown to be present in methanogenic sludge systems (58), wastewaters with recalcitrant compounds (59), and sugar-fed microbial fuel cells systems (60, 61). Additionally, members of the *Anaerolineae* class have been reported to contribute to the transformation of cellulose and hemi-cellulose to smaller carbon molecules such as lactate, formate, and acetate, even in adverse conditions, such as uranium-rich sediments, which was possible, given the presence of genes related to uranium tolerance (62). Members of the *Anaerolineae* classes can also be found in marine environments contributing to the re-cycling of dissolved organic matter and degrading carbohydrates (8, 63, 64).

Bacteria from the *Dehalococcoidia* class can perform anaerobic organohalide respiration, being repeatedly found in marine sediments at different worldwide locations, often with high relative abundances (65, 66). In fact, Krzmarzick et al. (29) investigated the role of *Dehalococcoidia* on chlorine cycle by establishing a correlation between their concentration and the concentration of organochlorine compounds, stating the pivotal role of these bacteria in the biogeochemical chlorine cycle (29). Additionally, a sulfur-oxidizing/reducing ability was reported by different authors, which could imply a role of *Dehalococcoidia* in the sulfur cycle of marine shallow surfaces (67).

Overall, members of the *Anaerolineae* and *Dehalococcoidia* contribute to the fermentation of sugars and fixation of carbon dioxide, participating in carbon cycling and constituting around 5%–25% of the bacterial communities detected in freshwater sediments from lakes and rivers (68, 69).

### Facultative or aerobic heterotrophy

Aerobic chemoheterotrophic metabolism can be found in the *Ardenticatenia, Ktedonobacteria*, *Tepidiformia*, and *Thermoﬂexia* classes. *A. maritima* (sole species of the *Ardenticatenia* class) is an aerobe that can also use ferric iron as an electron acceptor and tolerates high NaCl concentrations and temperatures, which can explain its abundance in iron-rich coastal hydrothermal fields (9). Members of *Ktedonobacteria* can grow in mesophilic or thermophilic conditions and have been reported to be dominant in coal-fire gas vents at 58°C, able to oxidize hydrogen and carbon monoxide for its metabolism (70). Additionally, *Ktedonobacteria* members have been found in steam vents from volcanoes (54). *Thermoﬂexia* members have been reported to have optimal growth at 72.5°C–75°C in microaerophilic conditions (1% vol/vol of O_2_), conditions usually found in hot springs sediments, where these bacteria may be found (24).

Within the aerobic organisms of the *Chloroflexota, Tepidiformia* class members, here represented by its single species *Tepidiforma bonchosmolovskayae*, are aerobic bacteria that can grow chemoorganoheterotrophically using different carbohydrates or volatile fatty acids and chemolithoautotrophically using FeCO_3_ as the electron donor, being usually found in hot springs (23).

*Chloroflexota* members exhibit several metabolisms, and despite being taxonomically divided into only eight classes, these bacteria can be found in extremely diverse environments adapted to different conditions, playing a role in the cycle of several elements such as carbon, sulfur, and halogens (71). The adaptability of *Chloroflexota* to different organic matter inputs allows their survival in adverse environments, a feature undoubtedly important for their application in the biotechnological industry.

## BIOTECHNOLOGICAL RELEVANCE

**Fig 4:**
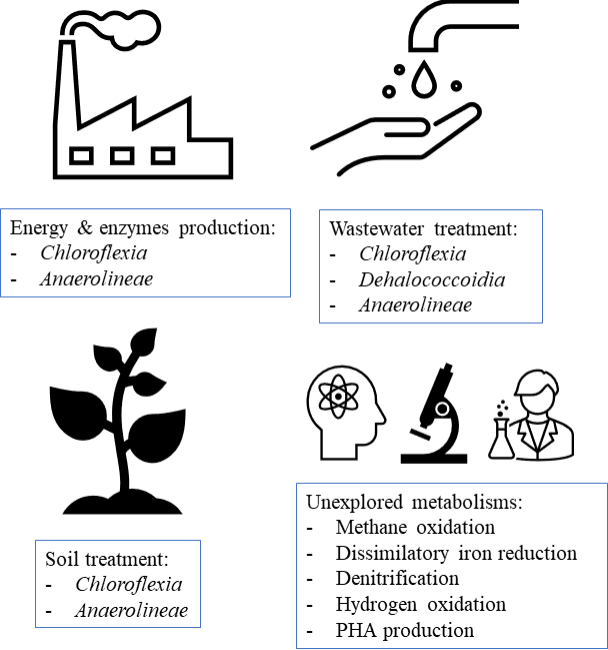
Applications of *Chloroflexota* members in different biotechnological areas.

The metabolic versatility of *Chloroflexota* and its natural occurrence in different habitats make this a very interesting group of bacteria to be used in several biotechnological applications, which can range from the production of chemical compounds to the degradation of contaminants, among many others (Fig. 4).

In fact, *Chloroflexota* bacteria can be found as sole contributors to certain applications or as an integrative of mixed microbial culture-based solutions. Regardless, this review aims to discuss *Chloroflexota* role in each technology, providing insights about the advantages of the utilization of these bacteria and prospecting the unexplored metabolisms that can be applied to the development of new biotechnological approaches.

### Production of enzymes and energy

Energy production is fundamental for human activities, powering essentially every aspect of daily life. Transportation, communications, and industrial production are paramount examples of the current energy demands. Complementarily, enzymes are widely applied in various industries (food and beverages, nutrition, textiles, cleaning products, and health and drugs sector) (72–74), ensuring product quality and stability while increasing production efficiency. The development of new approaches to produce enzymes and energy is imperative to decrease the environmental footprint of these activities, namely through the reduction of waste generation as well as water, energy, and raw materials demand.

Within *Chloroflexota*, the genus *Chloroflexus* has been a great source of enzymes. Shin et al. cloned and expressed in *Escherichia coli* the homodimer enzyme α-L-rhamnosidase (200 kDa MW) from *C. aurantiacus* and purified it as a soluble enzyme to use in the transformation of rutin (the bioflavonoid vitamin P) into isoquercitrin (flavonoid) (75). The obtained product has several important properties acting as an antioxidant, anti-inflammatory, anti-carcinogenic, antidiabetic, and anti-allergic agent (76), with the results displaying a natural ability from *C. aurantiacus* to effectively produce isoquercitrin. In fact, the purified α-L-rhamnosidase displayed the highest substrate-specific activity when compared with other isoquercitrin-producing enzymes. Moreover, isoquercitrin productivity from *C. aurantiacus*-derived α-L-rhamnosidase was almost two times higher than commercial α-L-rhamnosidase. Interestingly, this enzyme is also widely used in the industrial field for debittering citrus fruit juices, enhancement of wine aromas, and drug precursor production (77).

*C. aurantiacus* metabolic versatility was also investigated in the search for thermophilic alcohol dehydrogenases (78). These enzymes catalyze the regio- and stereo-selective reduction in aldehydes or ketones to primary or secondary alcohols, a process applied in several industrial-scale processes (79). Loderer et al. expressed this enzyme gene from *C. aurantiacus* in *E.coli* and reported its optimal temperature activity (70°C) and possible substrates, a relevant result given the industries’ demand for a larger diversity of well-characterized enzymes (78). The expressed enzyme showed high tolerance to ethylenediamine tetraacetic acid (EDTA), compared with other alcohol dehydrogenases, reflecting a stronger binding of the catalytic zinc ion, attributed to a more robust enzyme fold derived from a thermophilic host. Overall, the unique properties of the *Chloroflexota* enzyme (high-temperature activity, substrate versatility, and high tolerance to EDTA) broaden the enzyme’s applicability in various industrial processes, such as in the synthesis of fine chemicals, pharmaceuticals, or biofuels, benefiting from high-temperature stability, substrate flexibility, and robustness in the presence of chelating agents.

*Chloroflexia* bacteria are indeed a unique reservoir of new biocatalytic activities and the production of ene-reductase enzymes by *Chloroflexus aggregans* was investigated (80). These enzymes catalyze the asymmetric hydrogenation of alkenes and have collected great interest from academia and industries. Robescu et al. reported that the ene-reductases produced by *C. aggregans* were robust biocatalysts with high thermostability, presenting acceptable solvent tolerance and a wide range of optimal pH, which can be important for bigger-scale applications of this enzyme.

*Chloroflexi* bacterium, unclassified *Chloroflexota*, has been used to obtain ω-transaminases (ω-TA) highly reactive to aromatic amino donors/receptors at pH 8.5, 40°C, and showing affinity to cyclic substrates such as 1-Boc-3-piperidone (81). The enzymatic properties of this *Chloroflexota*-derived enzyme displayed good thermal stability, organic solvent tolerance, and broad substrate specificity. The ω-TA enzymes produce chiral amines with applications in the medical and fine chemical industries, such as the oral antihyperglycemic drug, sitagliptin. Therefore, the results of this study showed that *Chloroflexota* is a valuable source of catalysts for the asymmetric synthesis of these chiral amines from the corresponding aldehydes or ketones.

There are several examples of enzyme production with *Chloroflexota* bacteria, such as α-L-rhamnosidase, alcohol dehydrogenase, ene-reductase, and ω-transaminases. However, the reported studies have been using strains from the *Chloroflexia* class, which means that given the metabolic diversity presented by *Chloroflexota* members, there is still a tremendous amount of unexplored potential for enzyme production within this bacterial phylum, and further understanding of metabolic pathways and enzyme functions could help overcome this bottleneck.

Another relevant application is the utilization of *Chloroflexota* metabolic pathways to produce energy precursors, namely hydrogen or biogas. Both can be used as renewable sources of energy (82), and the development of new production approaches can contribute to the implementation of completely circular and bio-based solutions.

Production of hydrogen by *Chloroflexota* has been studied through the utilization of catalytic systems based on whole cells or organelles of photosynthetic bacteria specialized in the conversion of light energy into H_2_ (83). For this reason, Gogotov et al. studied the hydrogenases of several photosynthetic bacteria (including *C. aurantiacus*), proving the involvement of Ni (from the enzyme Ni-Fe active center) on the activation of molecular hydrogen and reporting a high denaturing factor resistance from these enzymes, which can be attributed to their thermophilic origin. Moreover, hydrogenase from *C. aurantiacus* was able to reversibly activate H_2_ at a high rate at more anaerobic conditions, in contrast with other studied hydrogenases that exhibited low activity under similar conditions. The ability to activate hydrogen efficiently at low redox potentials could be an evolutionary adaptation of *C. aurantiacus* to its natural habitat. In fact, hot springs often present anaerobic and reduced environments, and the microbial life in these locations has evolved mechanisms to optimally harness available resources, like hydrogen, for survival and growth. This feature can also be linked to potential biotechnological applications. For instance, enzymes that are active at low redox potentials and high temperatures could be advantageous in industrial processes involving hydrogen gas, such as in biohydrogen production or in enzymatic fuel cells.

Hydrogen production in photosynthetic microbial mats has been further expanded by biogeochemical and molecular studies reporting H_2_ production mainly under dark and anoxic conditions (84). The authors stated the importance of incorporating carbon during sunlight availability for dark production of H_2_ and concluded about the inexistence of competition between nitrogen fixation and H_2_ production. *Chloroflexales* participated in this process by being involved in the carbon capture and producing reduction equivalents for the dark production of H_2_, which states the importance of *Chloroflexota* bacteria in a mixed microbial culture approach specialized in coupling carbon capture with energy production (84).

Recent research reported the presence of *Anaerolineae* in the upgrading of antibiotic fermentation residue (AFR) to biogas (85). The study showed that the addition of Fe_3_O_4_ acted as a biostimulator for *Anaerolineae* activity, which enhanced methane production by as high as 48%. The contribution of *Anaerolineae* for the valorization of this protein-rich biosolid is another proof of the potential role of *Chloroflexota* bacteria in residue management strategies and energy production.

### Biodegradation technologies

Environmentally, microorganisms are the most important agents for the breakdown of organic pollutants or biodegradation, given their ability to use different harmful substances as carbon and energy sources (86). There are several examples of *Chloroflexota* microbial organisms’ contribution to biodegradation and decontamination technologies.

In the case of phenol-polluted environments, *Chloroflexota* can be used for their bioremediation. Phenolic compound pollution can be associated with wastewater discharges from several industries (87), with its removal being considered a priority by several countries and entities. Huang et al. assessed and stated the optimal conditions for phenol degradation (1.5 M of NaCl and 350 mg/L of phenol) using a mixed bacterial culture containing *Chloroflexus sp*. from a saline environment (88). Moreover, the authors studied the metabolic pathways related to phenol biodegradation and reported the importance of phenol hydroxylase, catechol 1,2-dioxygenase, and catechol 2,3-dioxygenase for this process. Furthermore, the dependence of this bacteria on ectoine and hydroxyectoine presence was established, contributing to improving bioremediation strategies in phenol-contaminated saline environments. Sanchéz-González et al. also stated the involvement of *Chloroflexales* in the degradation of phenol, highlighting the extremophile’s participation on the degradation process and the microbial community strategies to survive under severe environmental conditions (89). Overall, *Chloroflexales* bacteria can be considered effective phenolic degraders, and given their ability to thrive in anaerobic conditions, their utilization might be advantageous when treating certain types of industrial effluents. Moreover, their ability to establish synergies with other species can lead to more efficient degradation processes compared with microbes working in isolation. In comparison, genera like *Pseudomonas*, *Acinetobacter*, and *Sphingomonas* are more versatile and efficient in aerobic conditions and are widely used in industrial bioremediation processes (90, 91). Fungi offer a different mechanism through extracellular enzymatic degradation and are particularly effective against more complex pollutants (92). However, *Chloroflexales* might have a unique niche in phenol degradation, especially in anaerobic and extreme environments.

Zhang et al. studied the interactions of bacterial populations along sediment pollution gradients in shallow eutrophic lakes (93). The authors reported that *Chloroflexales* were among the dominant taxa at severe pollution concentrations, possibly contributing to photosynthesis and pollutant degradation, which further demonstrates the adaptability of *Chloroflexota* bacteria to adverse conditions and their importance in bioremediation technologies.

The organohalide respiration of *Dehalococcoides* presents great biotechnological interest in bioremediation applications, and Zanaroli et al. described the dechlorination capacity of members of this class, which was able to dechlorinate more than 75% of polychlorinated biphenyls (PCBs) in just 30 weeks (94). This extensive removal of pollutants is remarkable, given that PCBs are persistent organic pollutants and, due to bioaccumulation, are responsible for negative health effects on humans (95). In addition to being the first dechlorinator identified in marine sediments, the displayed dechlorination activity and specificity were more comprehensive than other bacteria described in the literature. Moreover, the activities of dechlorination took place under biogeochemical circumstances that closely mirror those found naturally in marine environments. This aspect is particularly important when considering the development of customized approaches for encouraging the *in situ* dechlorination of aged PCBs.

Padilla-Crespo et al. studied the environmental distribution of the genetic sequence encoding for the reductive dehalogenase, which catalyzes the dichloro elimination of 1,2-dichloropropane (a carcinogenic compound formerly used as industrial solvent) to propene (96). The authors reported gene sequences from different continents sharing high sequence identities (>98%), indicating that this enzyme is highly conserved or was recently disseminated. Moreover, *Dehalococcoides mccarty*, from the *Dehalococcoidia* class, appeared to be the major microbial contributor for this bioremediation process.

Organohalide-respiring *Dehalococcoidia* has been described, and its importance in bioremediation technologies has been stated before (16, 97). In fact, *Dehalococcoidia* presence has also been observed in industrialized estuaries sediments after extreme weather conditions (98). In this study, after Hurricane Harvey in 2017, the presence of several xenobiotic and polychlorinated compounds degrading microorganisms was correlated with sediment properties and contaminant concentrations of the estuary water.

*Chloroflexota* can also contribute to the biodegradation of polycyclic aromatic hydrocarbons (PAHs) and mutagenic/carcinogenic toxic compounds produced by incomplete combustion of fossil fuels. Specifically, in constructed wetlands, the low dissolved oxygen concentrations decrease the activity of PAHs-degrading microorganisms, such as *Pseudomonas aeruginosa* and *Pseudomonas putida*, which require aerobic conditions to perform aromatic hydrocarbon degradation (99). Therefore, the development of anaerobic approaches is important to enhance PAH removal for polluted sites with low available oxygen. Hence, Lu et al. reported the important role of *Anaerolineae* bacterium in the degradation of PAHs in an iron-enhanced anaerobic process, in which the metal presence functioned as an electron conduit to promote interspecies electron transfer between iron-reducing bacteria and *Anaerolineae* (100).

### Wastewater treatment technologies

The consistently high abundance of *Chloroflexota* bacteria in wastewater treatment systems illustrates their ecological role in nutrient transformation processes. These bacteria are often identified in nutrient (phosphorous and nitrogen) removal systems acting as anaerobic chemoorganotrophs with sugar fermentation abilities, being present in floccular biomass and/or in bulking-related representatives (101, 102). The understanding of Chloroflexota distribution and physiology is determinant to establish correlations between their ecology and operational issues in full-scale plants. Table 1 summarizes processes for wastewater treatment processes involving *Chloroflexota* bacteria.

**TABLE 1:** Wastewater treatment processes where the involvement of *Chloroflexota* bacteria was reported

Water type	Pollutant removal	Process type	Bacteria	Reference
Synthetic wastewater	97% of COD 97% of nitrogen	Submerged membrane bioreactors	*Chloroflexia, Anaerolineae*	(103)
Municipal wastewater	90% of DOC 92% of phosphorus 38% of nitrogen	Submerged membrane bioreactors	Unspecified *Chloroflexota*	(104)
Mainstream wastewater	90% of nitrogen	Anammox at low temperature	*Chloroflexales*	(105)
Aniline wastewater	80% of aniline 100% of nitrogen	Electro-enhanced sequencing batch reactor	*Anaerolineae*	(106)
Domestic saline wastewater	93% of COD	Membrane-aerated biofilm reactor	*Anaerolineae*	(107)
Saline wastewater	51.8% of COD	Constructed wetlands	*Anaerolineae, Dehalococcoides*	(108)
Thermal hydrolysis and anaerobic digestion wastewater	12% of COD 0.58 ± 0.06 g N/(L⋅d)	Partial nitritation-anammox	*Anaerolineae*	(109)
p-Fluoronitrobenzene (p-FNB) wastewater	100% of p-FNB	Bioelectrochemical degradation	*Anaerolineae*	(110)
Quinoline wastewater	83.5% of quinoline	Anaerobic degradation	*Anaerolineae*	(111)
Effluents of wastewater treatment plants	83% of nitrogen 43% of COD	Constructed wetlands	*Anaerolineae* Other *Chloroflexota*	(112)

As can be observed, these bacteria are present in several treatment processes, contributing to pollutant removal in a wide range of wastewater types, often as dissolved organic matter decomposers.

Specifically, *Chloroflexota* members have been detected in membrane bioreactors acting as soluble microbial product decomposers (103), this feature being previously demonstrated by the work of Miura et al., who correlated the carbohydrates degradation ability of the system with the concentration of *Chloroflexota* and stated an imperative *Chloroflexota* cell concentration above 10% to avoid membrane fouling (104), suggesting the ecological significance of *Chloroflexota* members in the reduction of membrane fouling in membrane bioreactors.

*Chloroflexota* bacteria were also found to be important in specific treatments of mainstream wastewaters. Lv et al. reported an abundance of *Chloroflexales* genera in flocculent sludge establishing symbiotic microbial interactions with anaerobic ammonia-oxidizing bacteria, which favored the annamox process in mainstream wastewaters at low temperatures, maintaining a nitrogen removal efficiency above 90% (105). Therefore, the coexistence of anaerobic ammonia-oxidizing bacteria and *Chloroflexales* appears to be an effective solution to overcome the challenges of anammox processes at low temperatures.

Other reports displayed the importance of *Anaerolineae* in the effectiveness of aniline and nitrogen removal (106), treatment of saline wastewater (107, 108), treatment of waste streams rich in ammonium and low on organic compounds (109), biodegradation of p-fluoronitrobenzene (110), complete anaerobic mineralization of quinoline (111), and denitrification processes (112).

### Soil treatment technologies

*Chloroflexota* bacteria have been reported in numerous approaches for soil treatment, contributing as producers of biodegradable organic matter and nutrients, and degrading recalcitrant molecules. They have been found in metal-contaminated soils of abandoned mines, and their concentration varied with the application of different phytostabilization techniques (113). In this study, among other reported bacteria, *Chloroflexota* organisms belonged to the *Chloroflexales* order and could perform anaerobic photoheterotrophy and, in dark aerobic conditions, chemoheterotrophy. Moreover, *Chloroflexota* members can present heavy metal resistance (114), which could imply their involvement in the decontamination of soils, especially in the reduction of heavy metal bioavailability and in the production of biodegradable organic matter.

Studies on the improvement of soil fertility also reported the relevance of *Chloroflexota*. Huang et al. successfully applied biochar to alleviate salt stress in a rice plantation field, to prevent crop production inhibition (115). The study reported the abundance of *Chloroflexota* (*Anaerolineaceae* family members) in the untreated soil, which decreased after the treatment with biochar mainly due to changes in the soil pH, suggesting that the regulation of the bacterial community is a key factor in achieving a satisfactory soil decontamination. Despite decreasing in concentration, *Chloroflexota* bacteria were still important in the transformation of inorganic carbon into organic matter and production of nutrients such as phosphorous and nitrogen, implying a central role in the symbiotic relationships established between soil bacteria, fungi, and plants. Indeed, in a study by Chen et al., *Chloroflexota* was demonstrated to be beneficial for soil treatment, acting as organic matter and nutrient producers and contributing to the decrease in N-loss bacterial activities. The authors studied a maize rhizosphere for 3 years and concluded that the presence of bacteria from the unclassified group *Chloroflexia* KD4-96 in soils contributed not only to plant growth but also to grain production (116).

*Chloroflexota* has also been demonstrated to participate in composting processes, specifically in the treatment of textile wastes (117). In this study, the authors mixed several textile waste concentrations with paper waste for composting and reported the presence of *Chloroflexota* bacteria participating in recalcitrant molecular degradation for mixtures containing 40%*–*60% of textile wastes.

*Anaerolineae* carbon fixation metabolism, via Arnon-Buchanan cycle (118), was also demonstrated to be important during the reduction of contaminants bioavailability by natural processes in anthropized freshwater sediments with high phosphorous concentrations and alkaline pH (119). The carbon fixation activity of *Anaerolineae* in surface sediments creates a flux of carbon that aids in the degradation of xenobiotic compounds by bacteria residing in the deeper, non-surface sediments. This dynamic illustrates a noteworthy symbiotic relationship, where the metabolic processes of surface-dwelling *Anaerolineae* enhance the capacity of deeper sediment bacteria to recover contaminated sites.

Hereabove, a variety of *Chloroflexota* metabolisms (carbon fixation, nutrient production, switch between anaerobic photoheterotrophy, and, in dark aerobic conditions, chemoheterotrophy) were highlighted, being mostly found in microbial communities for the treatment of soils and sediments. Their wide presence can be attributed to their resistance to adverse environmental conditions, and their role as primary producers may potentiate the activity of other microorganisms, establishing symbiotic relationships in these applications. However, it can be observed that most of the described applications that make use of *Chloroflexota* mainly report bacteria from the *Chloroflexia*, *Anaerolineae,* and *Dehalococcoides* classes (Table 2). Therefore, the discovery of new metabolic pathways and functions can act as a driving force for the development of biobased technologies containing *Chloroflexota* bacteria as the main players or as important contributors in mixed microbial systems.

**TABLE 2:** Reported applications for bacteria belonging to the *Chloroflexota* phylum^*a*^

Bacterial class	Application	Metabolism	Reference
*Chloroflexia*	H_2_ production	Anaerobic photoheterotrophic growth	(84)
*Anaerolineae*	Biogas production	Carbohydrate hydrolysis and proteolysis	(85)
*Chloroflexia*	Phenolic compound removal	Meta-cleavage pathway	(88, 89)
*Dehalococcoides*	PCB treatment	Dehalogenation	(94, 96)
*Anaerolineae*	Biodegradation of PAHs	Anaerobic heterotrophic growth	(100)
*Anaerolineae* *Chloroflexia*	Avoidance of membrane fouling in SMBR	Anaerobic heterotrophic growth	(103, 104)
*Anaerolineae* *Chloroflexia*	N-removal, biodegradation of pollutants	Anaerobic (photo)-heterotrophic growth	(105, 106, 110)
*Chloroflexia*	Reduction of heavy metal availability and production of biodegradable organic matter	Anaerobic photoheterotrophic and chemoheterotrophic growth	(114)
*Anaerolineae* *Chloroflexia*	Soil treatment	Anaerobic photoautotrophic and heterotrophic growth	(115, 116)
*Anaerolineae*	Reduction of contaminant bioavailability	Carbon fixation (Arnon-Buchanan cycle)	(119)

^*a*^ PCBs, polychlorinated byiphenyls; PAHs, polycyclic aromatic hydrocarbons; SMBR, submerged membrane bioreactor.

## PROSPECTIVE BIOTECHNOLOGICAL APPLICATIONS

*Chloroflexota* metabolic diversity continues to expand as scientific studies report previously unknown microbial processes. For instance, (120) described a new candidate of the *Chloroflexota* phylum (*Candidatus Chlorolinea photoalkanotrophicum*) with the ability to both perform phototrophy and oxidize methane and/or other small alkanes (120), which not only extends the known metabolic diversity of *Chloroflexota* but also offers exciting possibilities for biotechnological applications, especially in the areas of environmental remediation (biodegradation of alkanes), renewable energy (conversion of methane into biofuels), and carbon cycle management (carbon sequestration or transformation). In fact, the metagenomic-assembled genome of this species showed the ability of this species to perform several metabolisms, namely phototrophy, aerobic respiration, reduction of nitrites, carbon monoxide oxidation, and oxidation of carbon from methane and/or propane, and potentially fixate carbon using the pathway composed of hybridized components of the serine cycle and the 3-hydroxypropionate bi-cycle. These findings contribute to demonstrating the evolution and incorporation of new pathways into *Chloroflexota* promoted by horizontal gene transfer occurrences in natural habitats (120, 121).

The work of Kawaichi et al. described, for the first time, another interesting metabolism for a representative of the *Chloroflexota* phylum that could be biotechnologically viable (9). In this study, an isolate (belonging to the *Ardenticatenia* class) from a hydrothermal field with high iron concentrations was shown to perform dissimilatory iron reduction. Presenting a versatile metabolism with the ability to grow using oxygen, ferric iron, and nitrate as electron acceptors, this isolate also grows at different temperatures (30°C–75°C) and salt concentrations (0%–6% NaCl). This metabolic trait can play an important role in the bioremediation of subsurface environments contaminated with organic or metal contaminants.

Regarding the discovery of interesting metabolic pathways for *Chloroflexota* bacteria, there are also studies reporting the existence of newly found aerobic respiration and partial denitrification for *Anaerolineae* (122). In fact, despite most described *Anaerolineae* being classified as strict anaerobes, *Levilinea saccharolytica* displayed a branched aerobic respiration pathway, containing a NADH dehydrogenase, a succinate dehydrogenase, a heme-copper oxygen reductase, and a bd oxidase. Moreover, two nitrite reduction pathways were reported containing different nitrite reductases, able to reduce nitrite into nitric oxide or into ammonia. The presence of previously unknown pathways in *Anaerolineae* species suggests a wider physiological diversity than previously recognized for this *Chloroflexota* class, offering new opportunities for biotechnological applications: the aerobic and denitrification capabilities of these bacteria could play a role in converting nitrite into less toxic forms, contributing to the health of the soil microbiome, improving soil quality, and potentially enhancing crop growth. Moreover, the presence of enzymes like NADH dehydrogenase, succinate dehydrogenase, and oxygen reductases indicates their potential role in more efficient breakdown of organic matter in wastewater, leading to more effective and possibly faster treatment methods.

*A. maritima*, from the *Ardenticatenia* class, also presents a wide range of physiologies, which include aerobic respiration (containing enzymes from complex I, II, III, and three oxygen reductases), iron reduction, and a complete denitrification pathway composed of nitrate reductase, nitrite reductase, nitric oxide reductase, and nitrous oxide reductase (123). These findings could enhance the role of *Chloroflexota* bacteria in nitrogen removal technologies in wastewater treatment plants and, to a broader extent, the coupling of nitrogen, sulfur, and carbon cycles to be used in multipurpose bioreactors, which is of utmost technological relevance (124).

Hydrothermal systems, including terrestrial hot springs, contain diverse geochemical conditions that promote the discovery of novel metabolisms. Among the isolated bacteria from the different redox environments existing in an intertidal, anoxic, iron-, and hydrogen-rich hot spring that mixes with the oxygenated atmosphere and sulfate-rich seawater, there was a *Chloroflexota* member that not only presented the ability to fixate carbon, via Calvin cycle, but also had genes encoding for a hydrogenase, suggesting a lithoautotrophic capacity to oxidize hydrogen (125). This ability could be important in the utilization of these bacteria in waste-free biotechnological processes to directly convert electrical energy and inorganic substances into amino acids and other biologically active substances, contributing to sustainable bioproduction, where the goal is to minimize waste and maximize efficiency. Overall, the thermophilic nature of these *Chloroflexota* bacteria, thriving in hydrothermal systems, suggests that they possess enzymes and proteins that are stable and active at high temperatures. This thermophilic property can be particularly advantageous in industrial processes that operate at elevated temperatures, providing more robust and efficient systems, as in the production of bioenergy or in biocatalysis processes.

*Chloroflexota* from the *Ktenodobacteria* class (*Thermogemmatispora* sp. T81) has been demonstrated to persist using sub-atmospheric levels of H_2_ and CO (126). The authors reported that group 1 h [NiFe]-hydrogenases and type I carbon monoxide dehydrogenases were encoded in most of the studied reference genomes within the *Ktedonobacteriales*. Additionally, a meta-transcriptome study revealed that homologs of the group 1 f[NiFe]-hydrogenase of *Roseiflexus* species are highly expressed in geothermal microbial mats at night (55), possibly indicating atmospheric H_2_ oxidation within the photosynthetic *Chloroflexota* strains. These findings could also indicate a possible application of *Chloroflexota* bacteria in the treatment of syngas and industrial off-gas streams, often rich in hydrogen and carbon monoxide. In fact, the ability of *C. aurantiacus* to use hydrogen or sulfide for photoautotrophic growth (127, 128), combined with its capacity for polyhydroxyalkanoates (PHA) accumulation (129) and pigment production (130), could be explored as a circular process focused on carbon dioxide mitigation coupled with the production of value-added substances. PHA are natural polyesters with thermoplastic properties that are internally accumulated by bacteria as carbon and energy reserves, being considered interesting candidates for substituting traditional plastics (131). Recently, purple phototrophic bacteria have been described as suitable PHA-accumulating organisms (132), and its upscale challenges have been reviewed (133). There are several examples of phototrophic production of PHA using purple bacteria or cyanobacteria (134–138), and these bacteria have been reviewed as suitable phototrophic factories to couple resource recovery with the production of value-added substances (139). However, the PHA production capacity of phototrophic organisms of the *Chloroflexota* phylum remains mostly unexplored. In fact, phototrophy has been demonstrated to be present in seven bacterial phyla, and the development of omics methodologies can contribute to valuable metabolic, ecological, and physiological insights regarding photosynthesis and carbon fixation (5). A potential *Chloroflexota* member to develop such studies is *C. aurantiacus*, a phototrophic species that can use the 3-hydroxypropionate bi-cycle pathway for autotrophic carbon fixation, in which some intermediates of the pathway (such as acetyl-coA and propionyl-coA) are precursors for PHA accumulation (140). Studying PHA production in *Chloroflexota*, specifically *C. aurantiacus*, is crucial due to its unique metabolic pathways which may offer more efficient or varied PHA synthesis compared with well-studied purple bacteria. The photoautotrophic growth capabilities of *C. aurantiacus*, using hydrogen or sulfide, presents opportunities for sustainable, energy-efficient bioplastic production. In fact, exploring *Chloroflexota*’s PHA production can broaden the understanding of bioplastic synthesis and applications, complementing and extending the current knowledge derived from studies on purple bacteria. Furthermore, *Cloroflexus* potential to use CO_2_ fixation toward PHA production could contribute to new carbon capture technologies focused on bioplastic production, hence advancing new *Chloroflexota*-based bioprocesses for photoautotrophic biodegradable polymers production.

## CONCLUSION

The *Chloroflexota* phylum encompasses several biotechnologically interesting bacteria that can be frequently found in extreme environments and naturally adapted to unfavorable conditions due to the unique characteristics of its members. The resilience of these organisms can be attributed to their metabolic diversity, responding to site-specific requirements. In fact, *Chloroflexota* is present in several biotechnological approaches, including water treatment, pollutant biodegradation, and energy production. Due to the metabolic diversity and adaptability of the members belonging to this bacterial phylum, several interesting *Chloroflexota* enzymes have been isolated and studied, often reported as thermostable and highly efficient. However, the available literature reveals that several *Chloroflexota* microorganisms remain unexplored, which means that several bacterial functions and possibly interesting metabolisms are being overlooked. Therefore, further research is required to unlock the full potential of these microorganisms, especially within their photoautotrophic members, which could be useful for the development of CO_2_-negative technologies. The understanding of *Chloroflexota* role in natural cycles, under specific conditions, is fundamental for the development of new technologies based on these microorganisms.
